# Trade policy announcements can increase price volatility in global food commodity markets

**DOI:** 10.1038/s43016-023-00729-6

**Published:** 2023-04-10

**Authors:** Michael Brander, Thomas Bernauer, Matthias Huss

**Affiliations:** 1 Center for Comparative and International Studies, ETH Zurich, Zurich, Switzerland; 2 Zurich Knowledge Center for Sustainable Development & Informatics and Sustainability Research Group, University of Zurich, Zurich, Switzerland; 3 Faculty of Business and Economics, University of Basel, Basel, Switzerland

**Keywords:** Economics, Politics and international relations, Agriculture

## Abstract

Many countries use trade policy to insulate their domestic markets from price volatility. However, there is a widespread concern that such policies—particularly export restrictions—may amplify global price volatility, adversely affecting other countries. Here, using an original dataset on trade policy announcements on wheat and maize encompassing the food price crises of 2007–2008 and 2010–2011, we show that the announcement of trade policy changes can increase global price volatility. This effect applies not only to export restrictions but also to import liberalization measures and is most pronounced when markets are tight (stocks are low). Policymakers should work towards increasing stock levels to mitigate price volatility effects of trade policy changes. When markets are tight, export restrictions and import liberalizations should be avoided.

## Abstract (web summary)

Volatile food prices induce risk and uncertainty, challenging consumers and producers alike. This study uses historical trade policy and price datasets encompassing two recent food crises to investigate the impact that trade policies announced by specific countries may have on global agricultural markets.

## Main

In the wake of the 2022 war in Ukraine, prices in global agricultural markets, their volatility and their potential implications for global food security have, again, become a key topic on the international political agenda. As both Ukraine and Russia are major exporters of wheat, prices and price volatility have strongly increased in global agricultural markets with the start of the war^1^. Even before the start of the Russian invasion, however, prices of the world’s key staple foods had nearly reached levels last seen during the food price crises of 2007–2008 and 2010–2011 (ref. ^1^). During these earlier food price crises, the ensuing public and media debate focused on rising food price levels, but the political response quickly concentrated on food price volatility^2^, that is, fluctuations of food prices around their short-term trend. Policymakers around the world pushed for measures to limit food price volatility and stabilize prices^3^. This shared political priority was, therefore, also reflected in a dedicated target to ‘limit extreme food price volatility’ in the 2030 Agenda for Sustainable Development, adopted in 2015 (ref. ^4^).

The political focus on food price volatility can be explained by a shared belief among countries that such volatility is undesirable. In contrast, countries do not hold a common view on the issue of high food prices because they affect countries unevenly. Food price volatility, though, is widely seen as detrimental to producers and consumers alike, while high food prices have negative repercussions for consumers but benefit agricultural producers^5^. The welfare effect of high food prices hence depends on the sectoral composition of national economies. Where positive effects on agricultural producers outweigh adverse effects on food buyers, high food prices may, overall, have positive welfare and economic effects. The existing literature documents that the impact of high food prices on poverty and food security has indeed been uneven among and within countries^6–10^. In contrast, food price volatility induces price risk and uncertainty, which challenge both consumers’ and producers’ ability to make decisions that are optimal for their welfare^11–14^. For producers, it is more difficult to predict the potential returns of their investments, which can lead to reduced agricultural production^15^. Similarly, volatile food prices challenge consumers’ ability to appropriately plan and budget for their food expenses. While these implications of volatile food prices can negatively affect any country, the effects are particularly detrimental in the developing world, where consumers and producers have more limited options to hedge against price uncertainty^2,16–20^. Given the value of stable food prices for both consumers and producers, in contrast to differing effects of high food prices, it is hardly surprising that policymakers have been primarily interested in limiting domestic price volatility.

As has again become evident in the context of the war in Ukraine, national governments frequently resort to trade policy interventions as a means to stabilize domestic food prices and insulate domestic markets from volatile global market prices^19^. However, there is also widespread concern that such interventions can result in even more volatile global market prices, which affect other countries (beggar-thy-neighbour behaviour) and in turn transmit back to domestic markets^19^. These concerns are often voiced with respect to restrictive export policies^21,22^, such as the ban on wheat exports by India in 2007 and again in 2022. Increased global price volatility can also limit the effectiveness of such national trade policies in trying to achieve more stable domestic prices as global price volatility transmits back to domestic markets^23^. When national trade policy changes increase global price volatility, domestic policy objectives designed to stabilize domestic food prices can thus conflict with the shared global objectives agreed on in the 2030 Agenda for Sustainable Development.

To analyse the effects of announcements of trade policy changes on price volatility, we use an original dataset on such announcements for wheat and maize, covering the time period from 2005 to 2017. We focus on wheat and maize because of their importance in global agricultural production, consumption and commodity trade. These crops have in the past been subject to trade policy interventions, which implicates an adequate level of variation in the data, sufficient for distinguishing the relative influence of different directions and types of trade policy intervention. Our dataset allows us to analyse price volatility effects conditional on the type of trade policy announced, whereas previous work has focused either on one type of policy such as export restrictions^21^ or import levies^24^, or on aggregated measures of government protectionism^25^.

The price volatility estimation used in this paper is based on daily ranges in futures prices, that is, the difference between the highest and the lowest prices observed on a given day. This approach allows for more robust causal inference on the effects of trade policies and thereby substantially advances previous work, which was based on low-frequency price volatility estimates or trade policy data^21,25^. Trade policy interventions may, in principle, be both a consequence of changes in market fundamentals and a cause of such changes. However, announcements of trade policy changes on a given day, which we focus on, are very unlikely to be a response to a same-day change in market fundamentals, such as a production shock or same-day price volatility. The reason is that market shocks emanate from fast-paced individual decisions of many buyers and sellers, whereas it commonly takes several to many days for new market information to make it through policymaking apparatuses and result in a collective trade policy decision. This motivates our focus on daily data. Taken together, our paper provides important insights for policymakers weighing trade-offs between domestic and global policy objectives when using trade policy changes to insulate their domestic food markets.

## Trade policy types, stock levels and food price volatility

The extent to which trade policy interventions translate into price volatility depends on supply-and-demand elasticities^19^. For a global market of staple foods, these elasticities are generally assumed to be inelastic in the short term. On the production side, supply is inelastic in the short term due to the inherently lagged response of seasonal agricultural production, and demand is inelastic due to slow changes of dietary habits and, in developing countries, the dependence on staples for basic food security^19^. Our argument hence is that both import policies (demand shocks) and export policies (supply shocks) can affect world market price volatility.

Other factors may, however, moderate the effects of trade policies on price volatility. In particular, stockholding can affect the extent to which supply-and-demand shocks affect price volatility, which reflects the conceptual framework of the model of competitive storage^26–31^. Stocks dampen the effects of a given consumption or production shock on price volatility. In the presence of sufficient stocks, a given shock induces less uncertainty on future price developments, which translates into more limited price volatility effects.

Yet, stocks are more effective in reducing price volatility effects of positive supply and negative demand shocks, that is, increases in supply or reductions of demand, than of negative supply or positive demand shocks, that is, reductions of supply or increases of demand^32^. In the event of positive supply and negative demand shocks, stockholders respond by building up stocks, and the resulting price effect on the world market is dampened trough additional stock demand. In contrast, when a negative supply or positive demand shock affects world markets, the extent to which prices are moderated is limited by stock levels, that is, carry-overs from past seasons that can be released. Restrictive export policies and liberal import policies represent such negative supply and positive demand shocks, and we hence expect price volatility effects for these two trade policy types. Taken together, we expect global price volatility effects for restrictive export policies and liberal import policies, and pronounced effects in periods of low stocks.

## Results

### Wheat and maize trade policy changes in 2005–2017

**Fig. 1:**
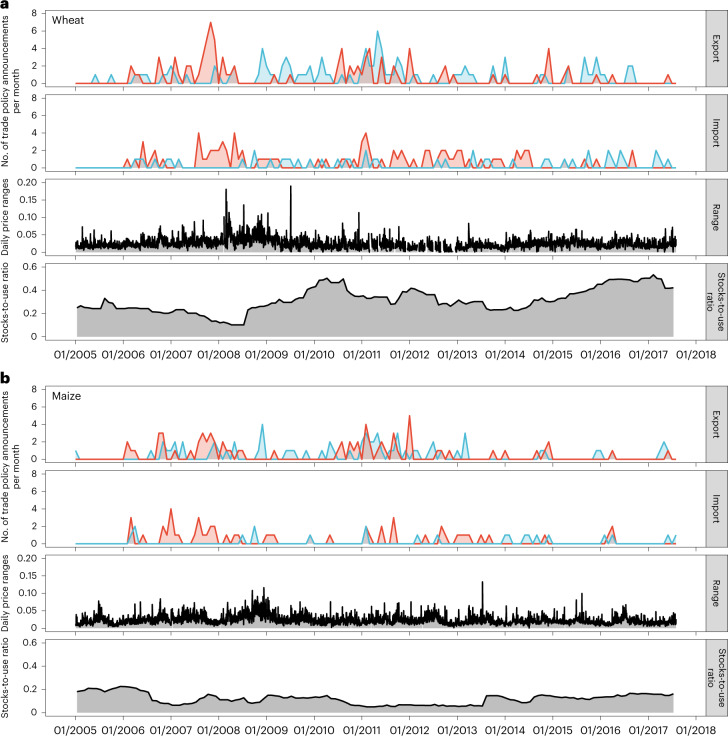
Frequency and direction of trade policy changes in 2005–2017. **a**,**b**, Trade policy announcements and price ranges (volatility) for wheat (**a**) and maize (**b**). For each commodity, the two upper rows show counts of monthly trade policy changes affecting exports (first row) and imports (second row). Red lines show trade policy changes that represent a negative supply or a positive demand shock, and blue lines show trade policy changes that imply positive supply or negative demand shocks. The two lower rows for each commodity show daily futures price ranges (third row) and the monthly stocks-to-use ratio (fourth row). Source data

In the observation period, the global trade of maize and wheat was subject to frequent trade policy announcements as well as changing patterns in price volatility. Figure 1 summarizes the number of trade policy announcements in each month during the observation period (see also Methods for details) and shows the daily price ranges as well as stocks-to-use ratios for each commodity.

Wheat was subject to a higher number of trade policy changes compared with maize. Although announcements of trade policy changes occur over the entire observation period, the number of announcements is particularly clustered in 2007 and 2011 for the two grains. The figure also indicates that price volatility is highly time varying and reaches a peak during 2008 and 2009, the time of the last major global food price crisis, suggesting that increases in volatility were preceded by more frequent trade policy changes in the months before. Likewise, stocks-to-use ratios change over time with low levels observed during 2007 and 2008 for both commodities and for maize again from 2011 to 2013. Maize stocks-to-use ratios are generally much lower than wheat stocks-to-use ratios.

Figure 1 further discriminates between export-related (first row) and import-related (second row) trade policy announcements, as well as the direction of trade policy changes in terms of their expected effect on world market supply and demand. Trade policies that represent a negative supply or a positive demand shock, for which we expect pronounced volatility effects, are shown with red lines. For example, a higher number of trade policy changes that imply a negative supply or a positive demand shock (red lines) is observed for 2007, whereas more trade policy changes that imply a positive supply or a negative demand shock (blue lines) occurred in 2009 (see also the base conditional autoregressive range model, CARRX, augmented with a dummy for 2007–2009 shown in Supplementary Table 1).

### Announcement day effects

To examine the price volatility effects of different types of trade policy intervention, we include policy dummy variables in our CARRX model and analyse their effects on announcement day price volatility (Methods). Price volatility on the announcement day refers to the day when the information of a trade policy event was first communicated and thus becomes available to market participants. If no futures contracts were traded on that day, the announcement day reflects the next date when futures contracts were traded again. We further examine periods of high and low stocks (suggesting tight global markets) from the stock-to-use ratio and classify a month as a low-stock period if the stocks-to-use level is below the first quintile during the observation period (reflecting a stocks-to-use ratio of 0.24 for wheat and of 0.07 for maize; Methods).

**Fig. 2:**
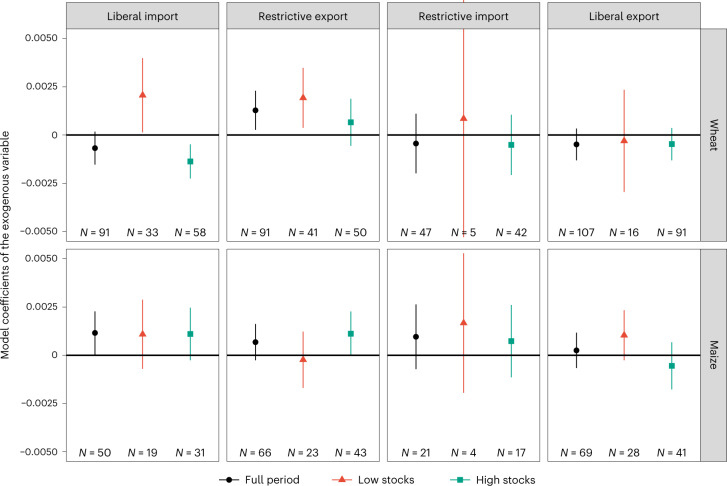
Effects of trade policy announcements on announcement day price volatility, contingent on stock levels. CARRX model coefficients of the exogenous variables (that is, policy dummies) by type of trade policy announced (positive export and import, as well as negative export and import trade policies), crop and the prevailing stock level at the time of the announcement. Coefficients show effect on global food price volatility by commodity on the announcement day. Data (points) are presented as coefficients of the exogenous variables (that is, policy dummies), and whiskers are 90% confidence intervals. Full regression results are shown in Supplementary Table 2. A robustness check for an alternative stocks threshold is shown in Supplementary Fig. 3. Source data

Our overall results displayed in Fig. 2 (black dots) show that the announcement of restrictive export policies leads to statistically significant increases in volatility for wheat (column 1) but not for maize, whereas the announcement of liberal import policies increases price volatility for maize (column 2). For wheat, the effects are contingent on prevailing stock levels at the time when a trade policy change is announced—when stocks are low (red triangles), liberal import policies also increase announcement day price volatility for wheat (column 1). For maize, stock levels do not appear to moderate the price volatility effects of trade policy announcements as indicated by similar coefficients for periods of low and high stocks.

Our results lend support to the hypothesis that both import and export policies can affect price volatility when they induce negative supply shocks (that is, restrictive export policies) or positive demand shocks (that is, liberal import policies), particularly in times of low stocks. Consistent with our hypothesis, we do not find significant effects for other types of trade policy, that is, announcements of liberal export policies or restrictive import policies (Fig. 2, columns 3 and 4), which reflect a positive supply shock and a negative demand shock, respectively.

As a robustness check for the role of countries’ trade shares, we restrict our sample to the top ten exporting and importing countries and re-run our analysis. The results remain qualitatively similar for maize and slightly accentuated for wheat, which is the more concentrated market (compare with Supplementary Fig. 1).

### Persistence of effects

From a policy perspective, the persistence of price volatility effects is of additional interest, as longer-term shocks are more likely to cause adverse effects on global commodity markets. To analyse the persistence of effects, we gradually extend the event window beyond the announcement day. Specifically, we consecutively add one or more days to the corresponding dummy variable in the conditional autoregressive range (CARR) model and re-estimate the model for each extended time window.

**Fig. 3:**
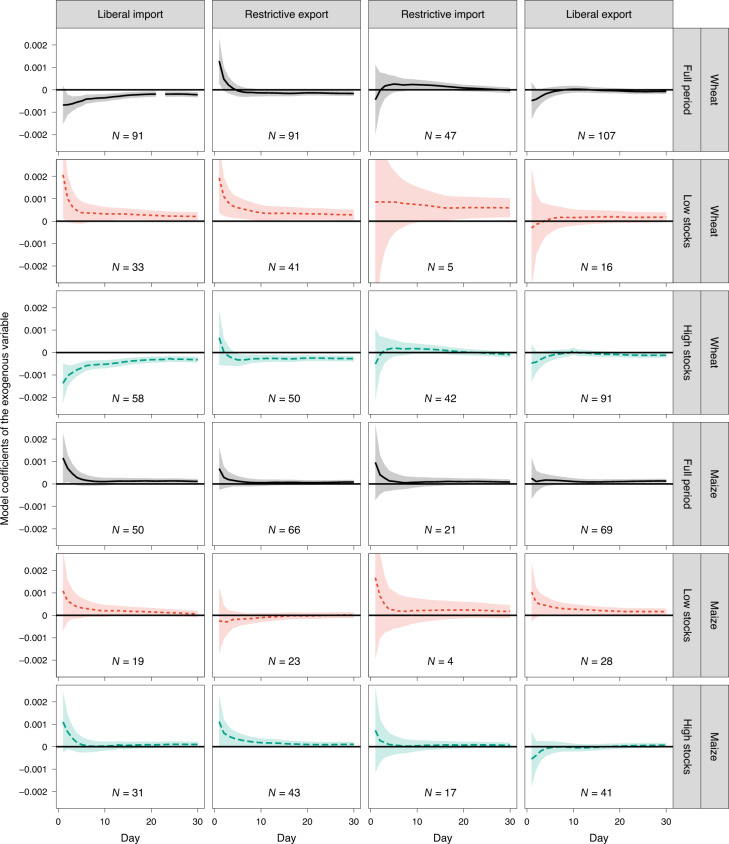
Persistence of abnormal price volatility by type of trade policy, crop and stock levels. Model coefficients by type of trade policy and crop. Each subpanel shows estimates by trade policy type (vertical) and by crop and stock level (horizontal). The *x* axis shows the number of days for the event window estimated. The *y* axis indicates effects on price volatility, expressed as the coefficients from model estimates. Data are presented as coefficients of the exogenous variables (that is, policy dummies), and ribbons are 90% confidence intervals. Full regression results are available from the authors upon request. A robustness check for major importers and exporters is shown in Supplementary Fig. 2, and a robustness check for an alternative stocks threshold is shown in Supplementary Fig. 4. Source data

Figure 3 shows that the effects extend beyond the announcement day and affect price volatility in the first ten trading days after the announcement before they tend towards zero as the event window is extended. The shocks tend to persist longer if stocks are low (red dotted line) compared with the overall estimation (black solid line) and compared with periods of high stocks (green dashed line). For example, the second tile (first row, second column) shows the effects of the announcement of export restrictions on wheat price volatility in the first 30 trading days after the announcement is made. Price volatility increases on the announcement day (that is, abnormal volatility is positive on day 1) and rapidly converges towards its long-run level as estimated by the endogenous model in the subsequent days (that is, cumulative abnormal volatility vanishes, indicating no persistent effects). When stocks are low, as shown in the tile below (second row, second column), abnormal volatility remains above zero for several days after the announcement, indicating more persistent price volatility effects beyond the announcement day.

It is important to note that our estimates show the average persistence of a single trade policy announcement in our observation period. However, announcements of trade policy changes are often clustered in a given period as shown in Fig. 1. A sequence of announcements, for example made by different countries within a month, may thus translate into longer periods of increased price volatility. In the case of wheat export restrictions, for example, more than one trade policy change was announced in 23 months in our observation period, which is the same number of months affected by one single trade policy change (Supplementary Table 3).

While our main estimation approach (CARR) is best suited to analyse (quasi-)causal effects of individual announcements, it is, by design, limited in its ability to estimate cumulative effects of several consecutive announcements. To illustrate how several trade policy changes could result in a longer period of increased price volatility, we also use a regression-based estimation reflecting a back-of-the-envelope approach. Specifically, we regress the count of monthly trade policy changes on monthly price variance for each type of trade policy, crop and in low- and high-stock periods. The monthly price variance is calculated as the sum of squared daily volatilities, estimated from the same set of observed daily price ranges used in the CARR model.

Consistent with our previous results, we find that the magnitude of the effects of liberal import and restrictive export policies on monthly wheat price volatility increases with the number of changes announced per month (Supplementary Table 4). These effects are accentuated when stocks are low. For example, we estimate that a single liberal import policy announcement increases monthly price variance of wheat by about 45%, whereas three liberal import policy announcements in the same month are estimated to increase monthly wheat price volatility by 135%. In the case of maize, the overall picture obtained from the results is similar to our previously presented results, although the effects of liberal import policies on monthly price volatility are not statistically significant. The latter may reflect that months with several trade policy changes are less frequent for maize compared with wheat. Overall, these results support the notion that the announcements of several trade policy changes in a given timespan—as is commonly observed—can translate into longer periods of increased price volatility, emphasising that the announcement of liberal import and restrictive export policy changes can cause adverse effects in global commodity markets.

## Discussion

National governments frequently use trade policy interventions in trying to stabilize domestic food prices and avert negative domestic welfare effects of volatile global food prices^2,3^. There is widespread concern that such policies—particularly export restrictions—might exacerbate global food price volatility, which then adversely affects other countries. The empirical evidence for such negative effects has, however, thus far remained rather thin. Hence the gap we have addressed in this paper. The main finding is that the announcement of trade policies that are expected to reduce available quantities on the world market—through either more restrictive export or more liberal import regimes—can amplify global price volatility. The price volatility effects materialize on the announcement day and remain persistent for approximately ten trading days.

We also find that these effects are more pronounced and persistent in periods of low stocks. When stocks are low, restrictive export policies increase global price volatility of wheat, but not of maize, and liberal import policies increase global price volatility of wheat and maize. We do not find any statistically significant increases in price volatility in times of high stocks. This result is consistent with our argument that stocks can mitigate the effects of a given trade policy shock on price volatility. However, stocks are more effective in mitigating policy shocks in the case of wheat compared with maize. For wheat, high stock levels curtail the effects not only of supply-side shocks (export restrictions) but also of demand-side shocks (liberalized import). However, stock levels do not appear to moderate the effect of trade policy announcements on maize price volatility. One potential explanation is that stocks-to-use ratios for maize are generally much lower compared with wheat, which may have limited the effectiveness of stocks in mitigating price volatility effects of trade policy announcements during the observation period.

One important contribution of our paper is the finding that liberal import policies can increase global price volatility too. This finding contrasts with the existing literature, which has argued, but not empirically tested, that liberal import policies “likely had little effect on world price volatility” (page 84 in ref. ^21^). Our findings (based on a much larger geographic scope) are broadly in line with a recent simulation study result showing that European Union import taxes increased maize price volatility in Argentina^24^.

Our findings also have implications for the broader literature on effects of trade policies on global food price levels. These studies, likewise, analyse effects of either export restrictions or protectionist measures in general, often concluding that trade policy interventions have strong effects on wheat prices but only small effects on maize price levels^33–36^. Our results, although specific to price volatility, are in line with this literature: we observe significant effects of export restrictions on wheat price volatility but not on maize price volatility. One potential interpretation is that the United States, which is the most important global exporter of maize by far, does not apply trade restrictions, which limits the influence other countries’ maize export restrictions may have on global price volatility^21^.

Our study analyses the announcement effects of trade policy changes on global food price volatility but does not empirically analyse the price volatility effects on the day an announced trade policy change is actually implemented. Our focus on announcements, rather than implementation dates, is motivated by the assumption that in efficient markets information on trade policy changes should be immediately reflected in market participants’ expectations and hence prices and volatility. In this case, the actual implementation of changes, which is rarely on the same date, does not provide new information. However, effects on the implementation day may differ, for example, in cases where there is uncertainty about whether or not an announced policy will actually be implemented or in cases where important implementation details remained unresolved when the change was first announced. While analysing such implementation day effects is outside the scope of this study, evaluating the price volatility effects of trade policy changes on their implementation day is an important topic for further research.

Likewise, further research could analyse the extent to which the strength and temporal reach of a trade policy announcement may moderate price volatility effects. Specifically, the implementation timing (immediate or with a time lag), anticipated duration of the measure and the expected rigour of the measures (for example, full export bans compared with partial bans) could presumably moderate price volatility effects. Furthermore, cross-price effects of policies affecting one commodity on price volatility of another commodity would be an interesting subject for further research. Another issue worth addressing in future research is whether excess speculation in futures markets^37^ may reduce the effects of trade policy changes on global food price volatility. Overall, future work could use machine learning or big data approaches to estimate the moderating effects of additional announcement, policy and contextual variables.

While our study analyses the effects of announced trade policy changes, we do not empirically address the mechanisms that cause such trade policy changes. Trade policy changes may be announced as a response to past global price volatility but could also result from other factors, such as national food security concerns. Trade policy may thus be endogenous to price volatility. As our study focuses on daily data, such endogeneity is unlikely to affect our main findings. It is empirically plausible to assume that trade policy changes on a given day are not the result of same-day price volatility: market shocks result from fast-paced individual decisions of many buyers and sellers, whereas it commonly takes much more time for new information from markets to percolate through policymaking apparatuses and end up with collective trade policy decisions. Yet, lead–lag relationships between the two variables are an interesting topic for further research.

These limitations notwithstanding, our results show that the announcement of trade policy changes, and restrictive export and liberal import policies in particular, can induce increases of global food price volatility on their announcement day and several consecutive days. When several such trade policy announcements are made within a relatively short time period (as is often the case in tight markets), they can amplify global price volatility quite substantially. How these global price volatility effects in turn affect food security and livelihoods of agricultural producers and poor consumers in developing countries is an important question for further research. The research presented in this article provides a foundation for further studies on the relationship between different types of trade policy announcement and short-term price volatility.

Our results highlight that policymakers should carefully consider the adverse effects of agricultural policy interventions on global price volatility. When markets are tight (characterized by low stocks), policymakers should avoid not only export restrictions but also import liberalization. For wheat—where we find strong moderating effects of stocks—policymakers should be especially careful when stocks are low. While current World Trade Organization rules permit export restrictions of agricultural products, they require countries to take potential impacts on importing countries into account, which is informed by this study. Our results highlight that impacts on other countries should be taken into account not only for export restrictions but also for import liberalization measures. In addition, our findings also imply that higher stock levels can minimize price volatility effects of trade policy changes and reduce their persistence, which highlights that increasing stock levels can be an effective policy instrument to mitigate price volatility effects of trade policy changes.

## Methods

We put our theoretical arguments to an empirical test on the basis of an original dataset on trade policy announcements from 2005 to 2017 and by estimating the storage-dependent effects of different types of trade policy on daily futures price ranges using a CARR model.

### Conceptual framework

We adopt a simple definition of agricultural trade policy. International trade refers to exchanges of commodities, such as goods and services, across national boundaries, whereas trade policies comprise the standards, goals, rules and regulations that govern such exchanges^38^. On the basis of this general concept, agricultural trade policy is defined here as (1) an actual or potential decision by a national government or an institution controlled by the national government that concerns (2) transboundary exchange in one or more agricultural commodities. The first component puts the focus on national trade policies. Trade policies by the European Union are included, as they can be understood as decisions pertaining to a group of sovereign countries. Multilateral trade agreements, such as those of the World Trade Organization, are not considered. The definition further excludes decisions of private sector traders but includes decisions by state-owned enterprises.

### Case selection

To study the influence of trade policy interventions on price volatility, we focus on wheat and maize. This choice is motivated by their importance in global agricultural production, consumption and commodity trade. These crops have in the past been subject to trade policy interventions, which results in an adequate level of variation in the data, which is sufficient for distinguishing the relative influence of different directions and types of trade policy intervention. We focus on a 12 year time period, from 2005 to mid-2017, which encompasses peaks in food prices observed for 2007 and 2008, as well as 2011, and periods of relatively stable or decreasing world market prices for grains since 2012. Currently available, comparable datasets only cover the time after 2008, for example the Global Trade Alert database^39^.

Although our dataset also includes one further commodity (rice), we do not include this commodity in our analysis. The Chicago Board of Trade (CBOT) futures prices are less representative as a proxy for spot market prices for rice compared with maize or wheat^40^.

### Empirical estimation

As we are interested in the announcement effects of trade policy changes (also referred to as ‘events’ in this paper) on food price volatility, we use an event study approach. Our empirical setting requires time series of daily volatilities. However, volatility (that is, the second moment of the return distribution) is generally unobservable and has to be estimated from observed prices. We estimate daily volatility for each crop of interest from the range-based approach^41^. Let *P*_*τ*_ be the price of an asset at time *τ*. The price range over an interval [*t* − 1, *t*], defined as

1 $$R_t = \max \left\{ {\ln \left( {P_\tau } \right)} \right\} - \min \left\{ {\ln \left( {P_\tau } \right)} \right\},$$

where $$\tau \in \left[ {t - 1,t} \right]$$,

is an unbiased estimator of volatility. Compared with standard return-based measures, which are based on the difference of close-to-close prices, the range-based estimator incorporates more information, as it also captures the intra-period (that is, within day) price movements that return-based volatility measures ignore.

For our empirical design, we use the CARR model^42^. The model is a variant of the (generalized) autoregressive conditional heteroskedasticity (ARCH/GARCH) family of models^43,44^, which are widely used for modelling time series of (conditional) volatilities. The CARR model, however, is based on ranges, rather than returns, which makes the model more informationally efficient^45,46^.

Although initially formulated as an autoregressive model, the CARR model can be extended to take additional explanatory variables into account (then termed CARRX). A CARRX model of order (*p*, *q*, *l*) is given by:

2 $$\begin{array}{l}R_t = \lambda _t\varepsilon _t,\\ \lambda _t = \omega + \mathop {\sum}\limits_{i = 1}^p {\alpha _iR_{t - i}} + \mathop {\sum}\limits_{j = 1}^q {\beta _j\lambda _{t - j}} + \mathop {\sum}\limits_{k = 1}^l {\gamma _kX_{t,k}} ,\end{array}$$

where *λ*_*t*_ denotes the conditional mean of the range, on the basis of all information up to time *t*, and *ε*_*t*_ is the shock to the range. The parameter *ω* characterizes the inherent uncertainty in the range, *α* describes the short-term impact of a previous shock, and *β* describes the long-term effect of past shocks to the range.

Exogenous variables are denoted by *X*_*t,k*_. The main explanatory variable used in this paper is trade policy changes, which we code as dummy variables that take on the value 1 on the announcement day of a trade policy event and are 0 otherwise. We construct one vector of dummy variables for each type of policy event. The parameter *γ* measures the impact of trade policy changes on conditional volatility, and *i*, *j* and *k* are indices. We assume asymptotic normality and obtain standard errors from the Hessian of the likelihood function with respect to the parameters. Similar approaches have been used in previous work^37,47^. The data analysis was done in MATLAB (version R2019b on a Windows 11 system). On the basis of our argument, a strong positive coefficient is expected for restrictive export and liberal import policies.

### Data on trade policy

The trade policy changes are identified through a media search and hand-coded in terms of their type and the direction of change. The coding procedure and indicators were developed by experts within our research consortium (Supplementary Notes).

The media search was done on the Factiva database and restricted to English-language articles on the Reuters Newsfeed, published between January 2005 and July 2017 (https://www.dowjones.com/professional/factiva). As we seek to assess the effects of trade policy on global food prices, measured at the Chicago Mercantile Exchange (CME), the Reuters Newsfeed allows us to capture the key trade policy events affecting the global markets. Search terms include keywords for classes of trade policy measures and all synonyms, singular and plural forms and, where applicable, verb forms, for example, quota and limit, duty and duties, suspension and suspend. The full search string is available in the code book in Supplementary Notes.

The media search resulted in 27,507 articles overall (including articles relating to rice, which is not part of this analysis as explained in ‘Case selection’). The date and time of publication as well as the standard reference number were automatically extracted from these articles, applying a text mining algorithm (using the R package ‘stringi’^48^, version 1.7.5). Apart from date and time, all indicators were hand-coded by a dedicated team at the Institute of Policy and Strategy for Agriculture and Rural Development, after a coding training workshop. The relevance of the article was assessed as the first step. As to be expected, the media search yielded a much smaller number of relevant articles; 1,165 articles were identified as relevant. As some of these relevant articles concerned several trade policy changes or affected more than one commodity, the total number of identified trade policy events is 1,737.

For the purpose of this analysis, the authors did additional hand-coding for all identified trade policy events to flag articles that present new information compared with previous articles on the same trade policy change. A total of 556 articles presented new information concerning maize or wheat trade policy interventions, which form the data used in this paper (for an overview of trade events by country and commodity, see Supplementary Table 5). Announcements referring to the same type of trade policy change that reaches the market on the same trading day (not necessarily the same announcement day, if no trading takes place) are counted as a single event, which explains slight differences in the number of observations among model specifications.

### Types of trade policy

For each article, the type of trade policy was coded, that is, whether it is a tariff measure or a non-tariff measure, as well as what kind. Tariffs are defined as “customs duties on merchandise import”^49^. Non-tariff measures are defined as “policy measures other than ordinary customs tariffs that can potentially have an economic effect on international trade in goods, changing quantities traded, or prices or both”^50^. We use the International Classification of Non-Tariff Measures developed by the Multi-Agency Support Team group to classify different types of non-tariff measure^50^. We summarize some very specific sub-classifications developed by the Multi-Agency Support Team in their higher-level groupings, thereby reducing the level of detail while maintaining the overall (aggregate) categories. The groupings, codings and respective descriptions are available in a separate codebook for trade policy measures (Supplementary Notes). Figure 1 provides an overview of the frequency and types of trade policy change announced in our observation period.

### Direction of change

We seek to assess the effects of trade policy changes contingent on whether they are expected to increase or decrease world market demand or supply. For the purpose of a simplified coding instruction, the direction of the reported change was coded. For example, if an export tax on maize is changed from 5% to 10%, the new policy is coded as ‘higher’. However, if an import quota on maize is changed from 1 million tonnes down to 0.5 million tonnes, the new policy is coded as ‘lower’. On the basis of these coded indicators, we developed a ruleset that shows for each type of trade policy and for each direction whether the trade policy change leads to higher or lower world market supply or higher or lower world market demand. For example, a higher export tax is expected to lower world market supply, while a lower import quota is anticipated to lower world market demand. The rules for classification of world market effects are shown in Supplementary Table 6.

### Grain price data

To proxy for global grain prices and their volatilities respectively, we use the prices of nearby futures contracts (that is, contracts with the shortest time to maturity) traded at the CBOT, which is part of the CME. This choice reflects the assumption that the analysis of food price volatility, as done in this paper, requires daily price observations. Such data are not available for (the generally unobservable) spot markets, in particular at the global level. The contracts traded at the CBOT are characterized by high trading volumes and usually provide the highest liquidity compared with other exchanges, in particular for wheat and maize (termed ‘corn’ at the CBOT). They are therefore typically the preferred contracts for global actors, even outside the United States, for the purpose of hedging against future price risks. These characteristics make them a suitable estimate for global grain prices, which are the basis for our estimates on daily price volatility.

The futures price data were obtained from the Thomson Reuters Datastream. To calculate our volatility measure, we gather the highest and lowest prices recorded by the exchange for each trading day. The sample period starts in January 2005, which coincides with the start of the data collected on trade policy announcements. The end of the sample period is March 2018 for each commodity. This enables us to analyse the volatility dynamics in the time following the last trade policy announcement in our dataset (June 2017).

### Stocks data

We identify periods of high and low stocks from the stock-to-use ratio, which is measured as the end of period stock level, divided by the period consumption. Specifically, we classify a month as a low-stock period if the stocks-to-use level is below the 0.2 percentile during the observation period (2005–2017). This threshold reflects a stock-to-use ratio of 0.24 for wheat and a ratio of 0.07 for maize. We use stocks-to-use data for the United States, which is compiled by the US Department of Agriculture and available at a monthly frequency from their World Supply and Demand Estimates report (http://apps.fas.usda.gov/psdonline). Data for global stock-to-use estimates are only available on an annual frequency. However, US stocks data may be a more relevant indicator for our analysis, given that price volatility is estimated here from the daily range of futures prices at the CME.

### Trade shares

As a robustness check we restrict our sample to major exporters and importers and re-run our analysis based on this reduced sample. Our data collection approach, based on a media search, is more likely to capture events of larger countries compared with events of smaller countries whose trade policy measures receive less media attention. We hence refrain from conducting a direct comparison (or weighting) of trade policy changes of smaller and larger exporters or importers as this could bias our results. This motivates our choice of a robustness check based on a restricted sample of only major importers and exporters (Supplementary Figs. 1 and 2). We use export and import quantity data from the database FAOSTAT (https://www.fao.org/faostat) to identify, for each commodity, the ten countries with the highest average export quantities and import quantities in our observation period. FAOSTAT contains data at the European Union member state level, while our trade policy dataset summarizes all European Commission and European Union member states events in one category. We hence assign all top ten European Union member states to the European Union category. At least one European Union member state is part of the ten major exporters and importers for each commodity, which implies that the European Union is among the major importers and exporters in all cases.

### Reporting summary

Further information on research design is available in the Nature Portfolio Reporting Summary linked to this article.

## Supplementary information

Supplementary Information Supplementary Figs. 1–4 and Tables 1–6, Codebook, Classification of Non-Tariff Measures, Operationalization of Supply and Demand Effects.

Reporting Summary

## Data Availability

The datasets generated during and/or analysed during the current study are available at 10.5281/zenodo.7075374. Further databases used in the study are (database numbers 1 and 2 are not publicly accessible): (1) the futures price data were obtained from the Thomson Reuters Datastream, (2) the media search was done on the ‘Factiva’ database, (3) stocks-to-use data were obtained from the *World Supply and Demand Estimates* report of the US Department of Agriculture (http://apps.fas.usda.gov/psdonline) and (4) export and import quantity data were obtained from the database FAOSTAT (https://www.fao.org/faostat). Source data are provided with this paper.

## Source data

Source Data Fig. 1 Statistical source data for Fig. 1 (frequency and direction of announced trade policy changes, and price ranges for wheat and maize).
Source Data Figs. 2 and 3 Statistical source data for Fig. 2 (effects of trade policy announcements on announcement day price volatility) and Fig. 3 (persistence of abnormal price volatility).

## Code availability

The source code is available from the corresponding author upon request.
