# Supplementary material for: Trade policy announcements can increase price volatility in global food commodity markets
Source: Nat Food. 2023 Apr 10;4(4):331–40. doi: 10.1038/s43016-023-00729-6 (PMC10154237; doi:10.1038/s43016-023-00729-6)
Supplement: Supplementary file 1 — Supplementary Figs. 1–4 and Tables 1–6, Codebook, Classification of Non-Tariff Measures, Operationalization of Supply and Demand Effects. [file 43016_2023_729_MOESM1_ESM.pdf]

nature food

Article https://doi.org/10.1038/s43016-023-00729-6

Trade policy announcements can increase price volatility in global food commodity markets

In the format provided by the authors and unedited

Supplementary Figures

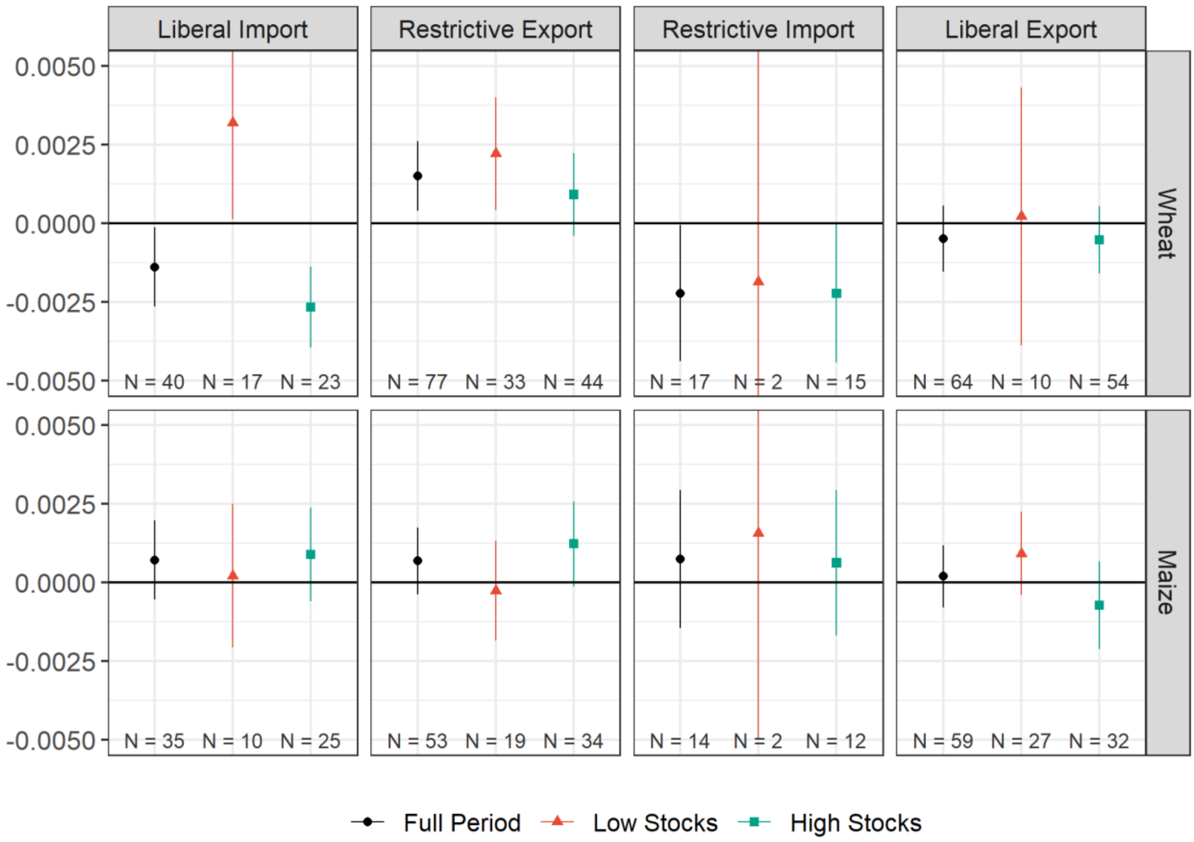

Supplementary Figure 1: **Effects of trade policy announcements on announcement day price volatility, contingent on stock levels – sub-sample of major importers/exporters**

The figure shows CARRX model coefficients of the exogenous variables (i.e., policy dummies) by type of trade policy announced (positive export and import, as well as negative export and import trade policies), crop, and the prevailing stock level at the time of the announcement. Analysis based on a sub-sample of major importing and exporting countries. Coefficients show effect on global food price volatility by commodity on the announcement day. Data (points) are presented as coefficients of the exogenous variables (i.e., policy dummies) and whiskers are 90% confidence intervals. Threshold for low stocks set at the 0.2 percentile. Full regression results are available from the authors upon request.

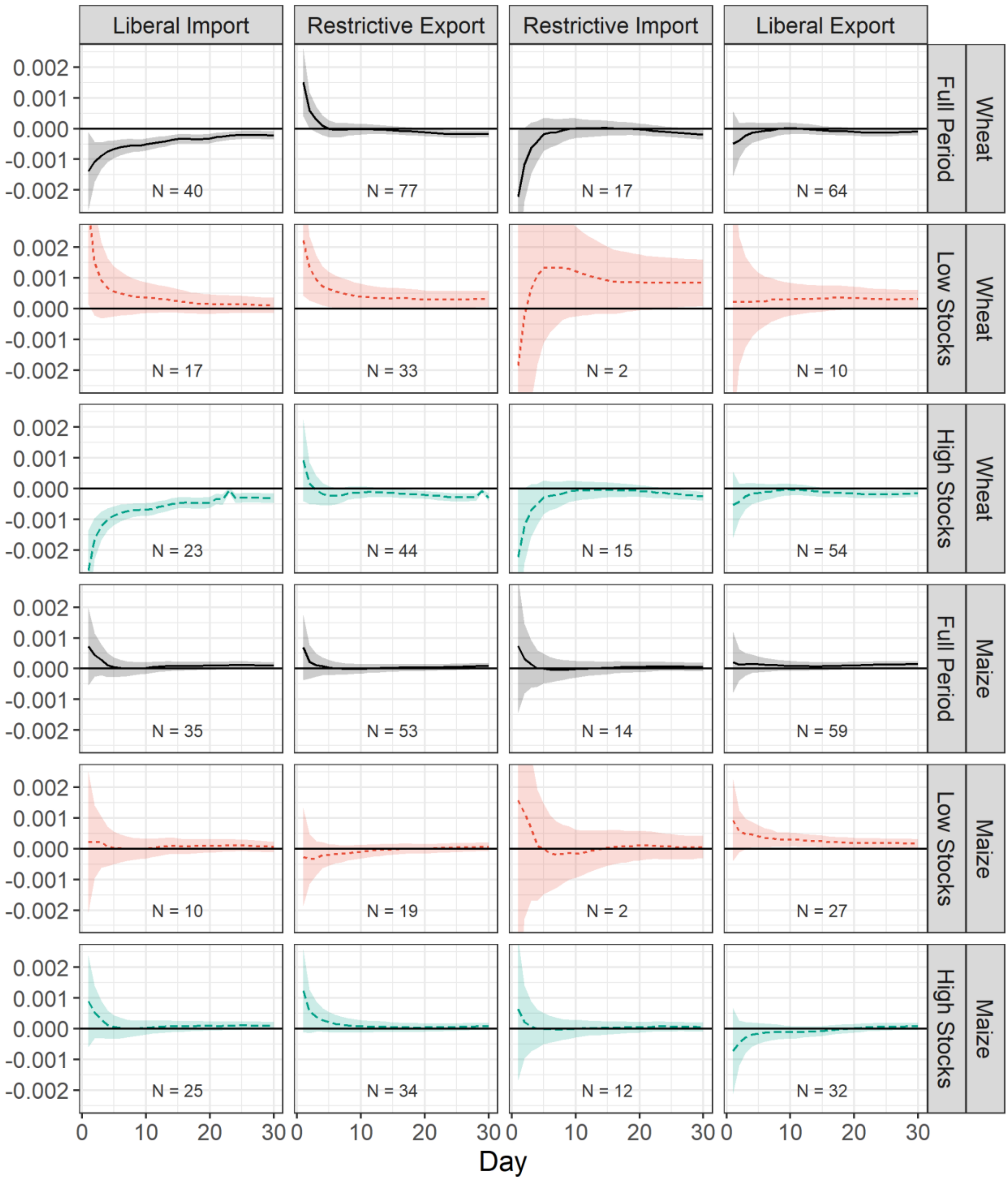

***Supplementary Figure 2:* Persistence of abnormal price volatility by type of trade policy, crop, and stock levels – sub-sample of major importers/exporters**

The figure shows model coefficients by type of trade policy and crop. Analysis based on a sub-sample of major importing and exporting countries. Each sub-panel shows estimates by trade policy type (vertical), and by crop and stock level (horizontal). The x-axis shows the number of days for the event window estimated. The y-axis indicates effects on price volatility, expressed as the coefficients from model estimates. Data (points) are presented as coefficients of the exogenous variables (i.e., policy dummies) and ribbons are 90% confidence intervals. Threshold for low stocks set at the 0.2 percentile. Full regression results are available from the authors upon request.

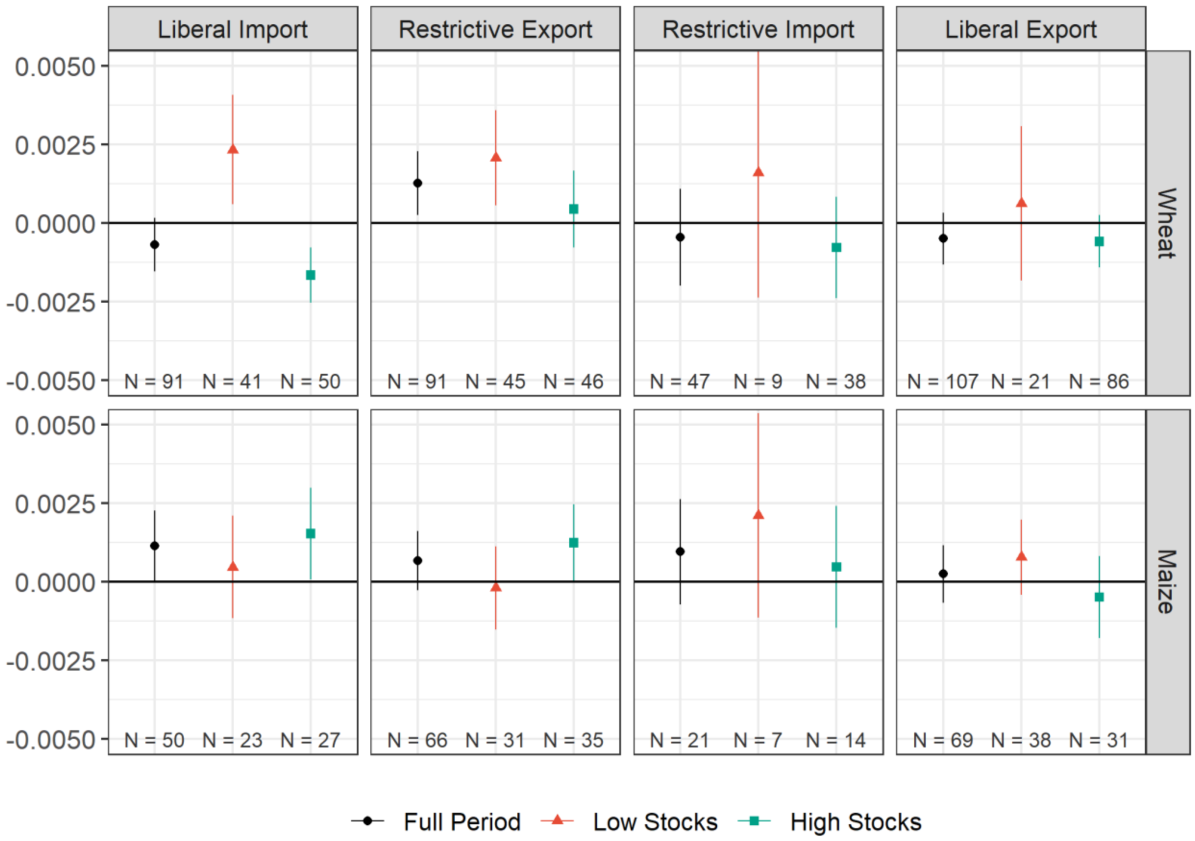

Supplementary Figure 3: **Effects of trade policy announcements on announcement day price volatility, contingent on stock levels - alternative stocks threshold**

The figure shows CARRX model coefficients of the exogenous variables (i.e., policy dummies) by type of trade policy announced (positive export and import, as well as negative export and import trade policies), crop, and the prevailing stock level at the time of the announcement. Analysis based on full sample. Coefficients show effect on global food price volatility by commodity on the announcement day. Data (points) are presented as coefficients of the exogenous variables (i.e., policy dummies) and whiskers are 90% confidence interval. Threshold for low stocks set at the 0.3 percentile. Full regression results are available from the authors upon request.

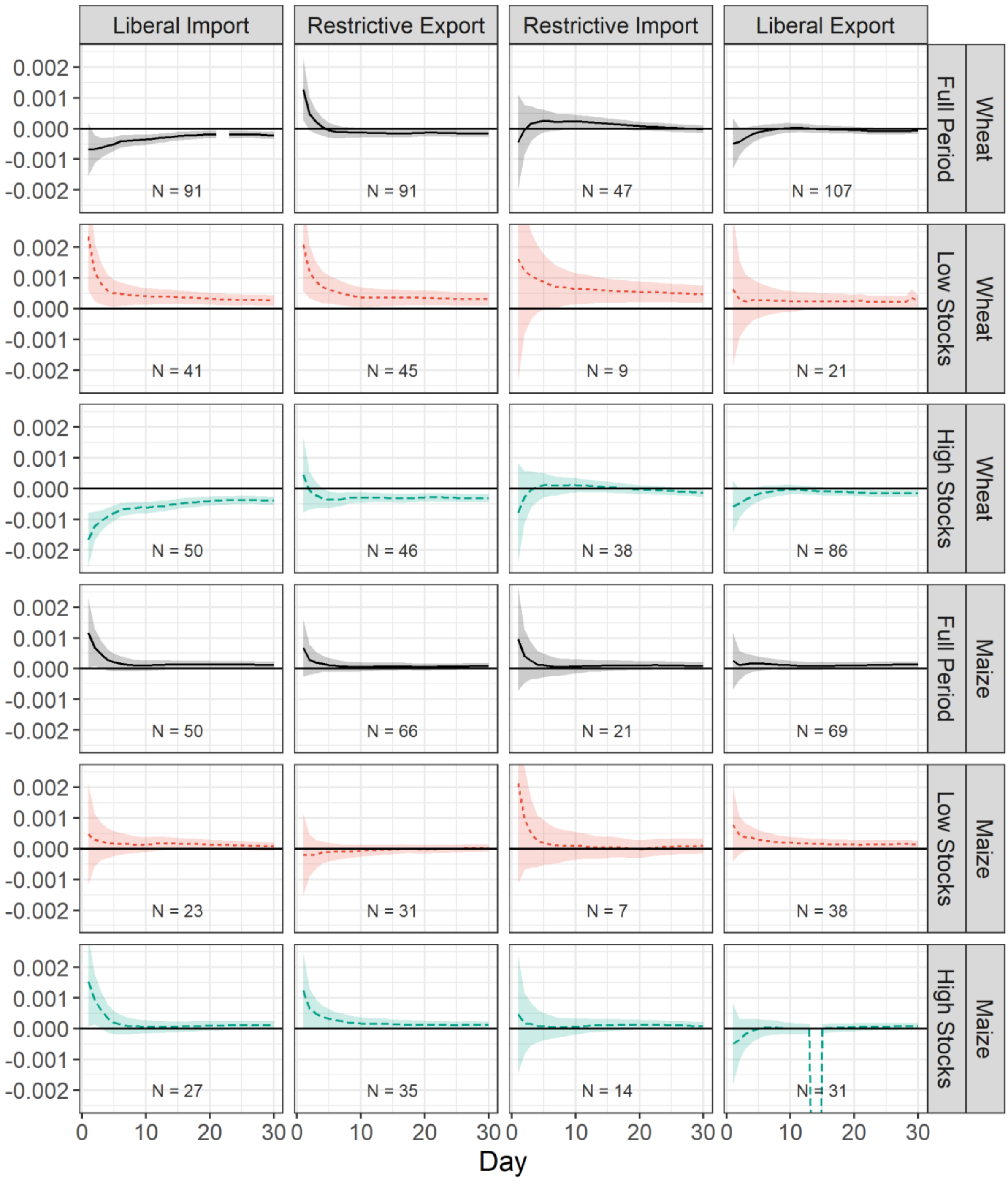

Supplementary Figure 4: **Persistence of abnormal price volatility by type of trade policy, crop, and stock levels – alternative stocks threshold**

The figure shows model coefficients by type of trade policy and crop. Analysis based on a sub-sample of major importing and exporting countries. Each sub-panel shows estimates by trade policy type (vertical), and by crop and stock level (horizontal). The x-axis shows the number of days for the event window estimated. The y-axis indicates effects on price volatility, expressed as the coefficients from model estimates. Data (points) are presented as coefficients of the exogenous variables (i.e., policy dummies) and ribbons are 90% confidence intervals. Threshold for low stocks set at the 0.3 percentile. Full regression results are available from the authors upon request.

Supplementary Tables

Overall, the impact of a prior shock to next day's volatility is similar for wheat and maize, as indicated by their alpha value. Likewise, the long-term effect of shocks is similar for wheat and maize, which is indicated by their beta value. The omega, alpha and beta coefficients reported in Table 2 remain almost identical in all augmented model specifications (i.e., augmented with policy dummy variables) presented in this paper. For the sake of brevity, we only report the coefficients for the exogenous variables added to the model. The complete results are available upon request from the authors.

		**omega**	**alpha**	**beta**	**2007/2009**
**Wheat**	Coefficient	0,0001	0,1459	0,8530	0,0002
	p-value	0,2224	0,0000	0,0000	0,2871
**Maize**	Coefficient	0,0004	0,1459	0,8374	0,0002
	p-value	0,0001	0,0000	0,0000	0,0814

Supplementary Table 1: CARR model estimates for the observation period and effects of years 2007-2009.

Table shows CARR model estimates with effect of spike year 7/2007-6/2009 as exogenous dummy variable. First row for each crop shows coefficients, second row shows p-values. We obtain standard errors from the Hessian of the likelihood function with respect to the parameters.

Crop	Trade Policy	Period	Coefficient	p-value	CI low (90%)	CI high (90%)
**Wheat**	Liberal Import	Full Period	-0.0007	0.1925	-0.0015	0.0002
		Low Stocks	0.0021	0.0780	0.0001	0.0040
		High Stocks	-0.0014	0.0110	-0.0023	-0.0005
	Restrictive Export	Full Period	0.0013	0.0377	0.0003	0.0023
		Low Stocks	0.0019	0.0415	0.0004	0.0035
		High Stocks	0.0007	0.3739	-0.0006	0.0019
	Restrictive Import	Full Period	-0.0004	0.6386	-0.0020	0.0011
		Low Stocks	0.0009	0.8199	-0.0053	0.0070
		High Stocks	-0.0005	0.5913	-0.0021	0.0011
	Liberal Export	Full Period	-0.0005	0.3323	-0.0013	0.0003
		Low Stocks	-0.0003	0.8503	-0.0030	0.0023
		High Stocks	-0.0005	0.3563	-0.0013	0.0004
**Maize**	Liberal Import	Full Period	0.0012	0.0886	0.0000	0.0023
		Low Stocks	0.0011	0.3189	-0.0007	0.0029
		High Stocks	0.0011	0.1833	-0.0003	0.0025
	Restrictive Export	Full Period	0.0007	0.2335	-0.0003	0.0016
		Low Stocks	-0.0002	0.7921	-0.0017	0.0012
		High Stocks	0.0011	0.1109	0.0000	0.0023
	Restrictive Import	Full Period	0.0010	0.3470	-0.0007	0.0026
		Low Stocks	0.0017	0.4473	-0.0019	0.0053
		High Stocks	0.0007	0.5214	-0.0011	0.0026
	Liberal Export	Full Period	0.0003	0.6498	-0.0007	0.0012
		Low Stocks	0.0010	0.1888	-0.0003	0.0023
		High Stocks	-0.0005	0.4593	-0.0018	0.0007

Supplementary Table 2: Effects of Import and Export Policy Announcements on Price Volatility in Full, Low Stocks and High Stocks Periods.

The table reports CARRX model coefficients of the exogenous variables (i.e., policy dummies) by type of trade policy (positive export and import, as well as negative export and import trade policy shocks), crop, and the prevailing stock level at the time of the announcement. Coefficients show effect on global food price volatility by commodity. We obtain standard errors from the Hessian of the likelihood function with respect to the parameters. Threshold for low stocks set at the 0.2 percentile.

	Liberal Import		Restrictive Export		Restrictive Import		Liberal Export	
	single events	multiple events	single events	multiple events	single events	multiple events	single events	multiple events
Wheat	36	25	23	23	33	7	40	26
Maize	26	11	23	18	15	3	31	16

Supplementary Table 3: Counts of Months with Single and Multiple Trade Policy Announcements (Events) in our Dataset.

Numbers show count of months where a single or multiple (more than 1) trade policy announcement was made per crop and per type of trade policy change.

Crop	Trade Policy	Period	Coefficient	SE	tStat	p-value	Intercept
**Wheat**	Liberal Import	Full Period	0.0007	0.0005	1.4354	0.1532	0.0058
		Low Stocks	0.0026	0.0007	4.0429	0.0001	0.0057
		High Stocks	-0.0011	0.0006	-1.7720	0.0783	0.0066
	Restrictive Export	Full Period	0.0007	0.0004	1.8132	0.0717	0.0058
		Low Stocks	0.0013	0.0005	2.7367	0.0069	0.0059
		High Stocks	-0.0003	0.0006	-0.5404	0.5897	0.0063
	Restrictive Import	Full Period	0.0002	0.0008	0.2079	0.8356	0.0062
		Low Stocks	0.0016	0.0025	0.6549	0.5135	0.0062
		High Stocks	0.0000	0.0008	-0.0017	0.9986	0.0062
	Liberal Export	Full Period	-0.0001	0.0004	-0.1783	0.8587	0.0063
		Low Stocks	0.0002	0.0011	0.1559	0.8763	0.0062
		High Stocks	-0.0001	0.0004	-0.2428	0.8085	0.0063
**Maize**	Liberal Import	Full Period	0.0007	0.0005	1.4663	0.1446	0.0053
		Low Stocks	0.0006	0.0007	0.7803	0.4364	0.0054
		High Stocks	0.0007	0.0006	1.1620	0.2470	0.0054
	Restrictive Export	Full Period	0.0003	0.0004	0.7504	0.4541	0.0054
		Low Stocks	-0.0001	0.0007	-0.1961	0.8448	0.0055
		High Stocks	0.0006	0.0005	1.0723	0.2852	0.0054
	Restrictive Import	Full Period	0.0009	0.0009	1.0798	0.2819	0.0054
		Low Stocks	-0.0007	0.0018	-0.4169	0.6773	0.0055
		High Stocks	0.0014	0.0010	1.4496	0.1491	0.0054
	Liberal Export	Full Period	0.0010	0.0004	2.3549	0.0198	0.0051
		Low Stocks	-0.0002	0.0006	-0.3724	0.7101	0.0055
		High Stocks	0.0019	0.0005	3.4482	0.0007	0.0050

Supplementary Table 4: Estimated Effects of Import and Export Policy Announcements on Monthly Price Variance in Full, Low Stocks and High Stocks Periods.

The table reports regression model coefficients of the exogenous variables (i.e., policy dummies) by type of trade policy, crop and stock level. Dependent variable is monthly price variance. Each line reflects a separate model. We use two-sided t-tests. No correction for multiple hypotheses tests was done. Stocks threshold at first quintile.

	Country	Total Number of Announcements	Announcements concerning Wheat	Announcements concerning Maize
**1**	Russian Federation	95	61	34
**2**	Ukraine	86	45	41
**3**	India	60	51	9
**4**	Argentina	58	33	25
**5**	European Union	38	24	14
**6**	China	32	9	23
**7**	Morocco	26	24	2
**8**	Pakistan	16	16	0
**9**	Brazil	14	10	4
**10**	Indonesia	10	7	3
**11**	Kazakhstan	9	6	3
**12**	Serbia	8	6	2
**13**	Taiwan, Province of China	8	3	5
**14**	Zambia	8	0	8
**15**	Korea, Republic of	7	5	2
**16**	Tanzania, United Republic of	7	3	4
**17**	Algeria	5	3	2
**18**	Canada	5	1	4
**19**	Philippines	5	5	0
**20**	Turkey	5	3	2
**21**	Viet Nam	5	1	4
**22**	Croatia	4	1	3
**23**	Kenya	4	1	3
**24**	Paraguay	4	2	2
**25**	Egypt	3	3	0
**26**	Guatemala	3	2	1
**27**	Iran, Islamic Republic of	3	3	0
**28**	Malawi	3	0	3
**29**	Mexico	3	0	3
**30**	Burundi	2	1	1
**31**	Cameroon	2	1	1
**32**	Romania	2	1	1
**33**	South Africa	2	2	0

**34**	Zimbabwe	2	1	1
**35**	Australia	1	1	0
**36**	Bangladesh	1	1	0
**37**	Bulgaria	1	1	0
**38**	Chile	1	1	0
**39**	Colombia	1	0	1
**40**	Iraq	1	1	0
**41**	Japan	1	1	0
**42**	Lebanon	1	1	0
**43**	Nepal	1	1	0
**44**	Peru	1	0	1
**45**	Saudi Arabia	1	1	0
**46**	Syrian Arab Republic	1	1	0

Supplementary Table 5: Counts of Trade Policy Events by Country in our Dataset.

Numbers show counts of total trade policy events recorded for the observation period.

Non-Tariff Measure Type		Market Effect	
Code	Name	Dir. Lower	Dir. High.
A	SANITARY AND PHYTOSANITARY MEASURES (SPS)	HD	LD
B	TECHNICAL BARRIERS TO TRADE (TBT)	HD	LD
C	PRE-SHIPMENT INSPECTION AND OTHER FORMALITIES	HD	LD
D	CONTINGENT TRADE-PROTECTIVE MEASURES	HD	LD
D1	Antidumping measure	HD	LD
D2	Countervailing measure	HD	LD
E	NON-AUTOMATIC LICENSING, QUOTAS, PROHIBITIONS AND QUANTITY-CONTROL MEASURES OTHER THAN FOR SPS OR TBT REASONS	NA	NA
E1	Non-automatic import-licensing procedures other than authorizations for SPS or TBT reasons	HD	LD
E2	Quotas	LD	HD
E3	Prohibitions other than for SPS and TBT reasons4	HD	LD
E311	Full prohibition (import ban)	HD	LD
E312	Seasonal prohibition	HD	LD
E313	Temporary prohibition, including suspension of issuance of licences	HD	LD
E314	Prohibition of importation in bulk	HD	LD
E315	Prohibition of products infringing patents or other intellectual property rights	HD	LD
E5	Export-restraint arrangement	HD	LD
E6	Tariff-rate quotas (TRQ)	LD	HD
F	PRICE-CONTROL MEASURES, INCLUDING ADDITIONAL TAXES AND CHARGES	HD	LD
F1	Administrative measures affecting customs value	HD	LD
F11	Minimum import prices	HD	LD
F12	Reference prices	HD	LD
F2	Voluntary export-price restraints (VEPRs)	HD	LD
F3	Variable charges	HD	LD
F4	Customs surcharges	HD	LD
F5	Seasonal duties	HD	LD
F6	Additional taxes and charges levied in connection to services provided by the government	HD	LD
F7	Internal taxes and charges levied on imports	HD	LD
G	FINANCE MEASURES	NA	NA
H	MEASURES AFFECTING COMPETITION	NA	NA
I	TRADE-RELATED INVESTMENT MEASURES	NA	NA
I1	Local content measures	HD	LD
I2	Trade-balancing measures	HD	LD
J	DISTRIBUTION RESTRICTIONS	HD	LD
K	RESTRICTIONS ON POST-SALES SERVICES	HD	LD

Non-Tariff Measure Type		Market Effect	
Code	Name	Dir. Lower	Dir. High.
L	SUBSIDIES (excluding export subsidies under P7)	LD	HD
M	GOVERNMENT PROCUREMENT RESTRICTIONS	HD	LD
N	INTELLECTUAL PROPERTY	HD	LD
O	RULES OF ORIGIN	HD	LD
P	EXPORT-RELATED MEASURES	NA	NA
P1	Export-license, -quota, -prohibition and other quantitative restrictions	NA	NA
P11	Export prohibition	HS	LS
P12	Export quotas	LS	HS
P13	Licensing- or permit requirements to export	HS	LS
P14	Export registration requirements	HS	LS
P19	Export quantitative restrictions (others)	HS	LS
P2	State-trading enterprises for exporting; other selective export channels	NA	NA
P3	Export price-control measures	HS	LS
P4	Measures on re-export	HS	LS
P5	Export taxes and charges	HS	LS
P6	Export technical measures	HS	LS
P7	Export subsidies	LS	HS

Supplementary Table 6: Operationalization of World Market Effects.

This table shows the operationalization of the expected world market supply and demand effects, depending on the direction of change coded. “Dir. Lower” means that the direction of change coded was “lower”, for example, an import quota was decreased. “Dir. Higher” means that a measure was increased. HD=Higher Demand, LD=Lower Demand, HS=Higher Supply, LS=Lower Supply. For some event categories, no world market supply and demand effect is known, for example, if the measure does not provide sufficient details (e.g. if only main category E is coded). In such case, NA is assigned.

Supplementary Notes

Codebook

The media search was done on the "Factiva" database and restricted to English-language articles on the "Reuters Newsfeed", published between January 2005 and July 2017 (see Chapter 3.4). The following search string was used:

(non-tariff or pre-shipment inspection* or trade-protective or antidumping or countervailing or licencing or licence* or quota* or prohibition* or ban or bans or banned or suspend* or restraint* or price-control* or tax or taxes or customs charge* or custom* or minimum price* or reference price* or export-price restraint* or variable charge* or customs surcharge* or duty or duties or internal tax or internal taxes or internal charge* or trade finance or trade financing or affecting competition or local content* or locali?ation or trade-balancing or distribution restriction* or post-sales service* or subsidies or subsidy or loan* or grant* or procurement* or rules of origin or rule of origin or quantitative restriction* or permit* or registration* or state-trading* or state trad* or re-export* or re-import*) and (import or imports or export or exports) and (wheat or maize or corn or rice)*

The following presents the coding guidance used by the research team, which can also serve to replicate the data collection and measurement.

General Rule:

- Type HELP if you don't know how to fill-out a specific cell and require assistance

a) Information Reference Number

Explanation: The variable contains the unique reference number assigned to each article. The number can be found at the very end of each media article, and looks for example like this "LBA0000020061009e2a90018a". Copy-paste the full string.

Coding:

- Copy-paste the information reference number, e.g. LBA0000020061009e2a90018a

b) Relevance of the Article:

Explanation: This variable pertains to whether the media article actually deals with agricultural trade policy. To be relevant, the article needs to deal with a) an actual or potential decision by a national government or an institution controlled by the national government, AND b) the decision affects the policy regime governing the transboundary exchange in rice, wheat or maize. For example, an article may describe ongoing negotiations in the World Trade Organization (WTO) on global trade liberalization. While that article may affect trade between countries, possible WTO decisions are not decisions by a national government or institution controlled by the national government. Hence, the article is considered irrelevant, and you would code: 0. If a media article is a duplication of a previous one coded, meaning that no new information is reported, code 0 and move on to the next article.

Note that government to government sales or tenders are only to be considered relevant if they mean a change in the national policy regime. For example, if Vietnam agrees to sell rice to Indonesia despite an export ban in place, it is relevant (code 1). However, if Vietnam announces a tender for rice exports, it is not considered a trade policy change and hence irrelevant (code 0).

Coding:

- Type 1 if the article deals with an agricultural trade policy
- Type 0 if the article does NOT deal with agricultural trade policy

Logic:

If you code 0, i.e. article is not relevant, move on to the next media article!

c) Originating jurisdiction

Explanation: This variable contains the information in which country or jurisdiction a decision affecting trade policy was made. For example, an article may inform that the Government of Russia has decided to stop all exports of wheat. In this case, you would code Russia by typing its shortcode: RU. In the event, that a trade policy change is specific to a small number of countries, type all their shortcodes separated by comma (,). For example, if Cambodia, Lao and Vietnam agree on a regional trade deal to decrease import tariffs for each other, code KH,LA,VN.

Required information: List of country/jurisdiction shortcodes available at: http://www.unece.org/cefact/locode/service/location

Coding:

- Type the two-character country/jurisdiction shortcode
- Type N/A if the information is not available in the media article

Logic:

If you code N/A, i.e. the article doesn’t say which country is the originator of a policy, then go to next media article

d) Date of the media article

Explanation: This variable pertains to the date of publication of the article as indicated on the article itself. You will have to fill-out three distinct cells, the first cell (column) captures the day of the month, the second cell captures the month, and the third cell the year. Coding example below applies to a possible date of a media article: 6th February 2013.

Coding:

- For the day of the month, specify the day in format dd (e.g. the 6th day of a month, type 06)
- For the month, type in format mm (e.g. if its February, type 02)
- For the year, type year in format yyyy (e.g. if its 2013, type 2013)

e) Goods affected

Explanation: This variable contains the information what products or goods are affected by the agricultural trade policy dealt with in the article. Affected products are those on which the transboundary exchange will have a potential influence. If more than one product is affected, yet not all of them are affected equally, you have to create separate entries. For example, if an article mentions that rice will be subject to a full export ban, and wheat will be subject to increased export tax, then create separate row entries for rice and maize.

Coding:

- Type W for wheat, if the trade policy affects wheat
- Type M for maize, if the trade policy affects maize/corn
- Type R for rice, if the trade policy affects rice
- If the agricultural trade policy concerns more than one product in the same way (e.g. maize and wheat), type all letters, e.g. MW or WMR (the order doesn't matter)
- If the agricultural trade policy concerns more than one product, but not all products are affected equally, copy-paste one additional row for each commodity the agricultural trade policy is related to, type the respective product code in the new fields, and continue coding for each.
- Type N/A if the information is not available in the media article

f) Tariff Measure

Explanation: This variable is a factor variable informing whether the trade policy measure is a form of import tariff or not. Import tariffs are "customs duties on merchandise imports". We distinguish between a) import tariffs on ad valorem basis (percentage of value), and b) import tariffs on a specific basis (e.g. $7 per 100 kgs.). For example, a news article may mention that the Government of Kenya is considering to increase its tariff on import of maize from 15% to 20%. In this case, you would code for an ad valorem import tariff and type 1.

Coding:

- If the trade policy is not an import tariff, code: 0
- If the trade policy is an import tariff at ad valorem basis, code: 1
- If the trade policy is an import tariff on specific basis, code: 2
- If the trade policy is an import tariff, but it is not specified if the tariff is ad valorem or specific, code: 3
- Type N/A if the information is not available in the media article

Logic:

If you code 1,2 or 3, skip the next indicator (only the next indicator, NOT going to the next article)

g) Non-Tariff Measure

Explanation: This variable pertains to the codes according to the International Classification of Non-Tariff Measures. All measures other than import tariffs, are classified according to this classification. The document contains definitions and examples for each of the possible trade policy measures. If you are uncertain, you can ask for assistance from the research team. For example, if a country decides to fully ban all exports for maize, that is classified as "Export prohibition". Hence the correct code is: P11

Required information: Own summary document "Summary: International Classification of Non-Tariff Measures – Codebook.docx"

Coding:

- Type the 3 to 4 character-digit combination according to the International Classification of Non-Tariff Measures, e.g. P11
- Type HELP in case you are uncertain and would like support from the research team
- Type N/A if the information is not available in the media article
- If more than one measure is concerned, create separate rows for each measure and continue with the next indicators for each measure.

h) Direction of Change

Explanation: This variable specifies the direction in which a trade policy is being changed. For example, if an export tax on maize is changed from 5% to 10%, the new policy is coded as "higher" (H). On the other hand, if an import quota on rice is changed from 1 Million tonnes down to 0.5 Million tonnes, the new policy is coded as "lower" (L). If a country decides to lift its export ban or import ban on rice, type lower (L). On the other hand, if a country introduces an export or import ban, type higher (H). If a country decides on stricter administrative procedures, like licensing requirements, type higher.

Coding:

- H: Type H for "Higher" if the direction is an increase
- L: Type L for "Lower" if the direction is a decrease
- HELP: Type HELP if some information is available, but you don't know how to code it
- N/A: Type N/A if the information is not given in the media article

i) Implementation Status

Explanation: The implementation status variable shows where a specific policy stands in the policy process. Three categories are distinguished: 1) Measure is under consideration, but not yet decided, 2) Measure is decided, but not yet in force, 3) Measure is in force.

Coding:

- 1: Type 1 if the measure is under consideration, but not yet decided
- 2: Type 2 if the Measure is decided, but not yet in force
- 3: Type 3 if Measure is in force
- N/A: Type N/A If the article doesn't include information on the status of implementation

j) Date of implementation

Explanation: The date of implementation is a variable pertaining to the date the trade policy change is enforced. You will have to fill-out three distinct cells, the first cell (column) captures the day of the month, the second cell captures the month, and the third cell the year. Coding example below applies to a possible date of implementation: 6th February 2013.

For example, if a media article mentions that an increase of import tariffs of the Government of Tanzania will be valid from 15th of April 2013, type 15 in the day cell, 04 in the month cell, and 2013 in the year cell. If a government announces that a partial export ban will be in place starting May 2013, type 5 in the month cell and 2013 in the year cell. In case a government decides upon altered export licensing requirements, and indicates they are effective immediately, type the date of the media article according to coding instructions.

If no day, month or year is given, but any other time indication (like "in autumn"), type OTHER.

Coding:

- For the day of the month, specify the day in format dd (e.g. the 6th day of a month, type 06)
- For the month, type in format mm (e.g. if its February, type 02)
- For the year, type year in format yyyy (e.g. if its 2013, type 2013)
- OTHER: If no specific day, month or year is given (e.g. “in autumn”), type OTHER
- type N/A if no information is given

k) End date

Explanation: For <u>temporary</u> trade policy changes, the end date is the date the documented change will be either withdrawn or fully replaced by a further change. For permanent measures, the measure needs to be coded as “permanent” without end date. For example, if a media article mentions that an increase of import tariffs of the Government of Tanzania will be valid until 31st of December 2013, type 31 in the day field, 12 in the month field, and 2013 in the year field. If a government announces that a partial export ban will be in place until December 2013, type 12 in the month field, and 2013 in the year field. In case a government decides upon altered export licensing requirements, which are meant to be permanent, type “P” in all three date fields.

All measures that do not explicitly specify that they are temporary, have to be coded as <u>permanent</u>.

Coding:

- For the day of the month, specify the day in format dd (e.g. the 6th day of a month, type 06)
- For the month, type in format mm (e.g. if its February, type 02)
- For the year, type year in format yyyy (e.g. if its 2013, type 2013)
- type P if the policy is implemented permanently
- type N/A if no information is given

l) Duration

Explanation: This variable captures the amount of time that a trade policy change is announced to remain in force. This variable has to be filled out if either the implementation date or the end date is missing. It hence has only to be coded in cases where implementation date or end date lack a specific day in their dates. Accordingly, for permanent measures, the field doesn’t have to be filled out.

Coding:

- If number of months is mentioned, type the number followed by M, e.g. 1M
- If number of weeks is mentioned, type the number followed by W, e.g. 4W

- If number of days is mentioned, type the number of days followed by D, e.g. 20D
- type N/A if no duration is provided

m) Information Source

Explanation: This variable identifies the source of the information reported in each media article. Two main categories of sources and their combinations are distinguished. A named source means that the source of information reported in a media article is attributed to an identified person or to an official communication from an institution (i.e. there is a name of a person, or a name of an institution). A government source means that the source is mentioned to be associated with the government. Example, a media article may state that China is will revise its import tariff on rice, according to government sources familiar with policy developments (without giving names), yet no named official confirms the information. In this case, the source is an unnamed government source, and you would type 3. If the source for the information is another newspaper or another media article, code 4 (unnamed non-government source).

Coding

- 1: Type 1 if Named Government Source: the source of the information is an identified government official or a named government institution
- 2: Type 2 if Named NON Government Source: Source of the information is an identified person or identified institution outside the government
- 3: Type 3 if Unnamed Government Source: Source of the information is an unnamed/anonymous government official or institution
- 4: Type 4 if Unnamed NON Government source: Source of the information is an unnamed/anonymous person or institution outside the government
- If more than one of the above source categories are given in an article, type each source that applies, separated by comma (,), for example: 1,3,4
- type N/A if no source information is given

Classification of Non-Tariff Measures

The following shows our own summary of the International Classification of Non-Tariff Measures from the MAST Group (Multi-Agency Support Team) as used for coding of the type of non-tariff measures in our dataset.

IMPORT NON-TARIFF MEASURES

A SANITARY AND PHYTOSANITARY MEASURES (SPS)

Measures that are applied to protect human or animal life from risks arising from additives, contaminants, toxins or disease-causing organisms in their food; to protect human life from plant- or animal-carried diseases; to protect animal or plant life from pests, diseases, or disease-causing organisms; to prevent or limit other damage to a country from the entry, establishment or spread of pests; and to protect biodiversity. These include measures taken to protect the health of fish and wild fauna, as well as of forests and wild flora. Note that measures for environmental protection (other than as defined above), to protect consumer interests, or for the welfare of animals are not covered by SPS.

B TECHNICAL BARRIERS TO TRADE (TBT)

Measures referring to technical regulations, and procedures for assessment of conformity with technical regulations and standards, excluding measures covered by the SPS Agreement. A technical regulation is a document which lays down product characteristics or their related processes and production methods, including the applicable administrative provisions, with which compliance is mandatory. It may also include or deal exclusively with terminology, symbols, packaging, marking or labelling requirements as they apply to a product, process or production method. A conformity assessment procedure is any procedure used, directly or indirectly, to determine that relevant requirements in technical regulations or standards are fulfilled; it may include, inter alia, procedures for sampling, testing and inspection; evaluation, verification and assurance of conformity; registration, accreditation and approval as well as their combinations.

C PRE-SHIPMENT INSPECTION AND OTHER FORMALITIES

C1 Pre-shipment inspection Compulsory quality, quantity and price control of goods prior to shipment from the exporting country, conducted by an independent inspecting agency mandated by the authorities of the importing country.

Example: A pre-shipment inspection of textile imports by a third party for verification of colours and types of materials is required.

D CONTINGENT TRADE-PROTECTIVE MEASURES

Measures implemented to counteract particular adverse effects of imports in the market of the importing country, including measures aimed at unfair foreign trade practices, contingent upon the fulfilment of certain procedural and substantive requirements.

D1 Antidumping measure

A border measure applied to imports of a product from an exporter. These imports are dumped and are causing injury to the domestic industry producing a like product, or to third countries' exporters of that product. Dumping takes place when a product is introduced into the commerce of an importing country at less than its normal value, generally where the export price of the product is less than the comparable price, in the ordinary course of trade, for the like product when destined for consumption in the exporting country. Antidumping measures may take the form of antidumping duties, or of price undertakings by the exporting firms.

Example: An antidumping duty of between 8.5 to 36.2% has been imposed on imports of biodiesel products from country A.

D2 Countervailing measure

A border measure applied to imports of a product to offset any direct or indirect subsidy granted by authorities in an exporting country where subsidized imports of that product from that country are causing injury to the domestic industry producing the like product in the importing country. Countervailing measures may take the form of countervailing duties, or of undertakings by the exporting firms or by authorities of the subsidizing country.

Example: A countervailing duty of 44.71% has been imposed by Mexico on imports of dynamic random access memory (DRAM) semiconductors from country A.

D3 Safeguard measures

A temporary border measure imposed on imports of a product to prevent or remedy serious injury caused by increased imports of that product and to facilitate adjustment. A country may take a safeguard action (i.e., temporarily suspend multilateral concessions) in respect of imports of a product from all sources where an investigation has established that increased imports of the product are causing or threatening to cause serious injury to the domestic industry that produces like or directly competitive products. Safeguard measures can take various forms, including increased duties, quantitative restrictions, and others (e.g. tariff-rate quotas, price-based measures, special levies, etc.).

E NON-AUTOMATIC LICENSING, QUOTAS, PROHIBITIONS AND QUANTITY-CONTROL MEASURES OTHER THAN FOR SPS OR TBT REASONS

Control measures generally aimed at restraining the quantity of goods that can be imported, regardless of whether they come from different sources or one specific supplier. These measures can take the form of non-automatic licensing, fixing of a predetermined quota, or through prohibitions. All measures introduced for SPS and TBT reasons are classified in chapters A and B above.

E1 Non-automatic import-licensing procedures other than authorizations for SPS or TBT reasons

An import-licensing procedure introduced, for reasons other than SPS or TBT reasons, where approval is not granted in all cases. The approval may either be granted on a discretionary

basis or may require specific criteria to be met before it is granted. Example: Imports of textile products are subject to a discretionary licence.

E2 Quotas

Restriction of importation of specified products through the setting of a maximum quantity or value that is authorized for import: No imports are allowed beyond those maximums. Example: A quota of 100 tons of fish where the importation can take place any time of the year and there is no restriction on the country of origin of the product.

E3 Prohibitions other than for SPS and TBT reasons

Prohibition on the importation of specific products for reasons other than SPS (A1) or TBT (B1) reasons.

E311 Full prohibition (import ban)

Prohibition without any additional condition or qualification Example: Imports of motor vehicle with cylinder under 1500cc are not allowed, to encourage domestic production.

E312 Seasonal prohibition

Prohibition of imports during a given period of the year: This is usually applied to certain agricultural products while the domestic harvest is in abundance. Example: Imports of strawberries are not allowed from March to June each year.

E313 Temporary prohibition, including suspension of issuance of licences

Prohibition set for a given fixed period of time unrelated to a specific season: usually for urgent matters not covered under the safeguard measures above. Example: Imports of certain fish are prohibited with immediate effect until the end of the current season.

E314 Prohibition of importation in bulk

Prohibition of importation in a large-volume container: Importation is only authorized if the product is packed in a small retail container, which increases per unit cost of imports. Example: Import of wine is allowed only in a bottle of 750 ml or less.

E315 Prohibition of products infringing patents or other intellectual property rights

Prohibition of copies or imitations of patented or trademarked products. Example: Import of imitation brand handbags is prohibited.

E5 Export-restraint arrangement

An arrangement by which an exporter agrees to limit exports in order to avoid imposition of restrictions by the importing country, such as quotas, raised tariffs or any other import controls. The arrangement may be concluded at either the government or industry level. Includes Voluntary export-restraint arrangements (VERs).

Example: A bilateral quota on export of motor vehicles from country A to country B was established to avoid sanction by the latter.

E6 Tariff-rate quotas (TRQ)

A system of multiple tariff rates applicable to a same product: The lower rates apply up to a certain value or volume of imports, and the higher rates are charged on imports which exceed this amount. Example: Rice may be imported free of duty up to the first 100,000 tons, after which it is subject to a tariff rate of $1.5 per kg.

F PRICE-CONTROL MEASURES, INCLUDING ADDITIONAL TAXES AND CHARGES

Measures implemented to control or affect the prices of imported goods in order to, inter alia, support the domestic price of certain products when the import prices of these goods are lower; establish the domestic price of certain products because of price fluctuation in domestic markets, or price instability in a foreign market; or to increase or preserve tax revenue. This category also includes measures other than tariffs measures that increase the cost of imports in a similar manner, i.e. by fixed percentage or by a fixed amount. They are also known as para-tariff measures.

F1 Administrative measures affecting customs value

Setting of import prices by the authorities of the importing country by taking into account the domestic prices of the producer or consumer. It could take the form of establishing floor- and ceiling-price limits; or reverting to determined international market values. There may be different price setting, such as minimum import prices or prices set according to a reference.

F11 Minimum import prices

Pre-established import price below which imports cannot take place. Example: A minimum import price is established for fabric and apparel.

F12 Reference prices

Pre-established import price which authorities of the importing country use as reference to verify the price of imports. Example: Reference prices for agricultural products are based on the farm-gate price, which is the net value of the product when it leaves the farm, after marketing costs have been subtracted.

F2 Voluntary export-price restraints (VEPRs)

An arrangement in which the exporter agrees to keep the price of the goods above a certain level: A VEPR process is initiated by the importing country and is thus considered as an import measure. Example: The export price of video cassette tapes is set higher in order to defuse trade friction with major importing countries.

F3 Variable charges

Taxes or levies aimed at bringing the market prices of imported products in line with the prices of corresponding domestic products: Primary commodities may be charged per total weight, while charges on processed foodstuffs can be levied in proportion to the primary product contents in the final product. Example: The target price for a seed is $700 per ton; since the world price is $500, there is a levy for $200. If the world price changed to $600, the levy would change to $100.

F4 Customs surcharges

An ad hoc tax levied solely on imported products in addition to customs tariff to raise fiscal revenues or to protect domestic industries. Example: Customs surcharge, surtax or additional duty.

F5 Seasonal duties

Duties applicable at certain times of the year, usually in connection with agricultural products. Example: Imports of fresh perry pears, in bulk from 1 August to 31 December may enter free of duty, while in other months, seasonal duties applied.

F6 Additional taxes and charges levied in connection to services provided by the government

Additional charges, which are levied on imported goods in addition to customs duties and surcharges and which have no internal equivalents.[7] They include: Custom-inspection, -processing and -servicing fees, merchandise-handling or -storing fees, tax on foreign exchange transactions, stamp tax, import licence fee, consular invoice fee, statistical tax ,tax on transport facilities, additional charges.

F7 Internal taxes and charges levied on imports

Taxes levied on imports that have domestic equivalents. For example, a tax on sales of products which are generally applied to all or most products.

G FINANCE MEASURES

Finance measures are intended to regulate the access to and cost of foreign exchange for imports and define the terms of payment. They may increase import costs in the same manner as tariff measures. Example: Payment of 100% of the estimated customs duty is required three months before the expected arrival of the goods to the port of entry.

H MEASURES AFFECTING COMPETITION

Measures to grant exclusive or special preferences or privileges to one or more limited group of economic operators. Example: A statutory marketing board with exclusive rights to control imports of certain grains, a canalizing agency with an exclusive right to distribute petroleum, a sole importing agency or importation reserved for specific importers regarding certain categories of goods.

I TRADE-RELATED INVESTMENT MEASURES

I1 Local content measures

Requirements to purchase or use certain minimum levels or types of domestically produced or sourced products, or restrictions on the purchase or use of imported products based on the volume or value of exports of local products. Example: In the production of automobiles, locally produced components must account for at least 50% of the value of the components used.

I2 Trade-balancing measures

Restrictions on the importation of products used in or related to local production, including in relation to the amount of local products exported; or limitations on access to foreign exchange

used for such importation based on the foreign exchange inflows attributable to the enterprise in question. Example: A company may import materials and other

J DISTRIBUTION RESTRICTIONS

Distribution of goods inside the importing country may be restricted. It may be controlled through additional license or certification requirements. For Example, restriction to limit the sales of goods to certain areas within the importing country.

K RESTRICTIONS ON POST-SALES SERVICES

Measures restricting producers of exported goods to provide post-sales service in the importing country. Example: After-sales servicing on exported TV sets must be provided by a local service company of the importing country.

L SUBSIDIES (excluding export subsidies under P7)

Financial contribution by a government or public body, or via government entrustment or direction of a private body (direct or potential direct transfer of funds: e.g. grant, loan, equity infusion, guarantee; government revenue foregone; provision of goods or services or purchase of goods; payments to a funding mechanism), or income or price support, which confers a benefit and is specific (to an enterprise or industry or group thereof, or limited to a designated geographical region). Example: The government provides producers of chemicals a one-time cash grant to replace antiquated production equipment.

M GOVERNMENT PROCUREMENT RESTRICTIONS

Measures controlling the purchase of goods by government agencies, generally by preferring national providers. Example: A government office has a traditional supplier of its office equipment requirement, in spite of higher prices than similar foreign suppliers.

N INTELLECTUAL PROPERTY

Measures related to intellectual property rights in trade: Intellectual property legislation covers patents, trademarks, industrial designs, layout designs of integrated circuits, copyright, geographical indications and trade secrets. Example: Clothing with unauthorized use of trademark is sold at much lower price than the authentic products.

O RULES OF ORIGIN

Rules of origin cover laws, regulations and administrative determinations of general application applied by government of importing countries to determine the country of origin of goods. Rules of origin are important in implementing trade policy instruments such as antidumping and countervailing duties, origin marking and safeguard measures. Example: Machinery products produced in a country are difficult to fulfil the rules of origin to qualify for the reduced tariff rate of the importing country, as the parts and materials originate in different countries.

EXPORT NON-TARIFF MEASURES

P EXPORT-RELATED MEASURES

Export-related measures are measures applied by the government of the exporting country on exported goods.

P1 Export-license, -quota, -prohibition and other quantitative restrictions

Restrictions to the quantity of goods exported to a specific country or countries by the government of the exporting country for reasons such as a shortage of goods in the domestic market, regulating domestic prices, avoiding antidumping measures or for political reasons.

P11 Export prohibition

Prohibition of exports of certain products. Example: Export of corn is prohibited because of a shortage in domestic consumption.

P12 Export quotas

Quotas that limit value or volume of exports. Example: An export quota of beef is established to guarantee adequate supply in the domestic market.

P13 Licensing- or permit requirements to export

A requirement to obtain a licence or a permit by the government of the exporting country to export products. Example: Exports of diamond ores are subject to licensing by the Ministry.

P14 Export registration requirements

A requirement to register products before being exported (for monitoring purposes). Example: Pharmaceutical products need to be registered before being exported.

P19 Export quantitative restrictions (others)

P2 State-trading enterprises, for exporting; other selective export channels

P21 State-trading enterprises, for exporting Enterprises (whether or not State-owned or -controlled) with special rights and privileges not available to other entities, which influence through their purchases and sales the level or direction of exports of particular products (See also H1). Example: An export monopoly board, to take advantage of terms of sale abroad; a marketing board, to promote for export on behalf of a large number of small farmers.

P3 Export price-control measures

Measures implemented to control the prices of exported products. Example: Different prices for exports are applied from the same product sold in the domestic market (dual pricing schemes).

P4 Measures on re-export

Measures applied by the government of the exporting country on exported goods which have originally been imported from abroad. Example: Re-export of wines and spirits back to the producing county is prohibited. The practice is common in cross-border trade to avoid imposition of domestic excise tax in the producing country.

P5 Export taxes and charges

Taxes collected on exported goods by the government of the exporting country: they can be set either on a specific or an ad valorem basis. Example: An export duty on crude petroleum is levied for revenue purposes.

P6 Export technical measures

Export regulations referring to the technical specification of products and conformity assessment systems thereof: Control over the quality or other characteristics of products for export. Example: Exports of processed food products must be inspected for sanitary conditions; or certification required by the exporting country

P7 Export subsidies

Financial contribution by a government or public body, or via government entrustment or direction of a private body (direct or potential direct transfer of funds: e.g. grant, loan, equity infusion, guarantee; government revenue foregone; provision of goods or services or purchase of goods; payments to a funding mechanism), or income or price support, which confers a benefit and is contingent in law or in fact upon export performance (whether solely or as one of several conditions), including measures illustrated in annex I of the Agreement on Subsidies and Countervailing Measures and measures described in the Agreement on Agriculture. Example: All manufacturers in country A are exempt from income tax on their export profits.
