# Supplementary material for: Trade policy announcements can increase price volatility in global food commodity markets
Source: Nat Food. 2023 Apr 10;4(4):331–40. doi: 10.1038/s43016-023-00729-6 (PMC10154237; doi:10.1038/s43016-023-00729-6)
Supplement: Supplementary file 2 — Reporting Summary [file 43016_2023_729_MOESM2_ESM.pdf]

nature research

Corresponding author(s): Matthias Huss

Last updated by author(s): Mar 1, 2023

Reporting Summary

Nature Research wishes to improve the reproducibility of the work that we publish. This form provides structure for consistency and transparency in reporting. For further information on Nature Research policies, see our Editorial Policies and the Editorial Policy Checklist.

Statistics

For all statistical analyses, confirm that the following items are present in the figure legend, table legend, main text, or Methods section.

n/a	Confirmed	
☐	☒	The exact sample size (n) for each experimental group/condition, given as a discrete number and unit of measurement
☒	☐	A statement on whether measurements were taken from distinct samples or whether the same sample was measured repeatedly
☐	☒	The statistical test(s) used AND whether they are one- or two-sided<br>*Only common tests should be described solely by name; describe more complex techniques in the Methods section.*
☐	☒	A description of all covariates tested
☐	☒	A description of any assumptions or corrections, such as tests of normality and adjustment for multiple comparisons
☐	☒	A full description of the statistical parameters including central tendency (e.g. means) or other basic estimates (e.g. regression coefficient) AND variation (e.g. standard deviation) or associated estimates of uncertainty (e.g. confidence intervals)
☐	☒	For null hypothesis testing, the test statistic (e.g. F, t, r) with confidence intervals, effect sizes, degrees of freedom and P value noted<br>*Give P values as exact values whenever suitable.*
☒	☐	For Bayesian analysis, information on the choice of priors and Markov chain Monte Carlo settings
☒	☐	For hierarchical and complex designs, identification of the appropriate level for tests and full reporting of outcomes
☐	☒	Estimates of effect sizes (e.g. Cohen's d, Pearson's r), indicating how they were calculated

Our web collection on statistics for biologists contains articles on many of the points above.

Software and code

Policy information about availability of computer code

Data collection	The media search was done on the "Factiva" database. Search results were downloaded as PDF files. The full search string is available in Supplementary Information SI-3. Date, time, and standard reference number of each article were automatically extracted using text mining (with R package "stringi", version 1.7.5).
Data analysis	The data analysis was done in MATLAB, version R2019b on a Windows 11 system. The source code is available from the authors upon request.

For manuscripts utilizing custom algorithms or software that are central to the research but not yet described in published literature, software must be made available to editors and reviewers. We strongly encourage code deposition in a community repository (e.g. GitHub). See the Nature Research guidelines for submitting code & software for further information.

Data

Policy information about availability of data

All manuscripts must include a data availability statement. This statement should provide the following information, where applicable:

- Accession codes, unique identifiers, or web links for publicly available datasets
- A list of figures that have associated raw data
- A description of any restrictions on data availability

The datasets generated during and/or analysed during the current study are available in the Zenodo repository: https://doi.org/10.5281/zenodo.7075374
Further databases used in the study are (Databases number 1 and 2 are not publicly accessible):
1) The futures price data was obtained from the Thomson Reuters Datastream
2) The media search was done on the "Factiva" database
3) Stocks-to-use data was obtained from the United States Department for Agriculture's World Supply and Demand Estimates report (http://apps.fas.usda.gov/

psdonline)
4) Export and import quantity data was obtained from the database “FAOSTAT” (https://www.fao.org/faostat)

Field-specific reporting

Please select the one below that is the best fit for your research. If you are not sure, read the appropriate sections before making your selection.

☐ Life sciences ☒ Behavioural & social sciences ☐ Ecological, evolutionary & environmental sciences

For a reference copy of the document with all sections, see nature.com/documents/nr-reporting-summary-flat.pdf

Behavioural & social sciences study design

All studies must disclose on these points even when the disclosure is negative.

Study description

quantitative, longitudinal study

Research sample

Trade policy announcements cover wheat and maize in the time period from 2005 until mid 2017. This choice of crops is motivated by their importance in global agricultural production, consumption and commodity trade. Timeframe encompasses food price crises of 2007/2008, as well as 2011. Dataset identifies announcements based on the Reuters Newsfeed, which allows us to capture the key trade policy events affecting on global markets.

To proxy for global grain prices, and their volatilities, respectively, we use the prices of nearby futures contracts (i.e., contracts with the shortest time to maturity) traded at the Chicago Board of Trade (CBOT), which is part of the Chicago Mercantile Exchange. This choice reflects the assumption that the analysis of food price volatility, as done in this paper, requires daily price observations. The contracts traded at the CBOT are characterised by high trading volumes, and usually provide the highest liquidity compared to other exchanges, in particular for wheat and maize (termed “corn” at the CBOT). They are therefore typically the preferred contracts for global actors, even outside the US, for the purpose of hedging against future price risks. These characteristics make them a suitable estimate for global grain prices, which are the basis for our estimates on daily price volatility. The futures price data was obtained from the Thomson Reuters Datastream.

We use stocks-to-use data for the United States, which is compiled by the United States Department for Agriculture (USDA) and available at monthly frequency from their World Supply and Demand Estimates report. Data for global stock-to-use estimates are only available on an annual frequency. Additionally, United States stocks data may be a more relevant indicator for our analysis, given that price volatility is estimated here from the daily range of futures prices at the Chicago Mercantile Exchange.

Sampling strategy

The trade policy changes are identified through a media search and hand-coded in terms of their type and the direction of change. The coding procedure and indicators were developed by experts within our research consortium. Supplementary Information SI-2 provides the full codebook.

Data collection

The media search to identify trade policy announcements resulted in 27’507 articles overall. The date and time of publication as well as the standard reference number were automatically extracted from these articles, using text mining. Apart from date and time, all indicators were hand-coded. Relevance of the article was assessed as the first step. As some of these relevant articles concerned several trade policy changes or affected more than one commodity, the total number of identified trade policy events is 1’737. For the purpose of this analysis, the authors did additional hand-coding for all identified trade policy events to flag articles that present new information as compared to previous articles on the same trade policy change. 556 articles presented new information concerning maize or wheat trade policy interventions, which form the data used in this paper.

Timing

The trade policy and stocks dataset covers the timeperiod between January 2015 and July 2017. For futures prices, the sample period starts in January 2005, which coincides with the start of the data collected on trade policy announcements. The end of the sample period is March 2018 for each commodity. This enables us to analyse the volatility dynamics in the time following the last trade policy announcement in our dataset (June 2017).

Data exclusions

Although our dataset also includes one further commodity (rice), we do not include this commodity in our analysis. Only a small fraction of rice is traded internationally, and CBOT futures prices may be less representative as proxy for spot market prices for rice as compared to maize or wheat.

Non-participation

No participants were involved in this study.

Randomization

Random allocation was not applicable for this study. The explanatory variable (different types of trade policy announcements) is outside the control of researchers and cannot be allocated randomly.

Reporting for specific materials, systems and methods

We require information from authors about some types of materials, experimental systems and methods used in many studies. Here, indicate whether each material, system or method listed is relevant to your study. If you are not sure if a list item applies to your research, read the appropriate section before selecting a response.

Materials & experimental systems

n/a	Involved in the study
☒	☐ Antibodies
☒	☐ Eukaryotic cell lines
☒	☐ Palaeontology and archaeology
☒	☐ Animals and other organisms
☒	☐ Human research participants
☒	☐ Clinical data
☒	☐ Dual use research of concern

Methods

n/a	Involved in the study
☒	☐ ChIP-seq
☒	☐ Flow cytometry
☒	☐ MRI-based neuroimaging
